# Amygdala‐related electroencephalogram neurofeedback as add‐on therapy for treatment‐resistant childhood sexual abuse posttraumatic stress disorder: feasibility study

**DOI:** 10.1111/pcn.13591

**Published:** 2023-12-22

**Authors:** Naomi B. Fine, Liat Helpman, Daphna Bardin Armon, Guy Gurevitch, Gal Sheppes, Zivya Seligman, Talma Hendler, Miki Bloch

**Affiliations:** ^1^ School of Psychological Sciences, Faculty of Social Sciences Tel‐Aviv University Tel Aviv Israel; ^2^ Sagol Brain Institute Tel‐Aviv, Wohl Institute for Advanced Imaging Tel‐Aviv Sourasky Medical Center Tel‐Aviv Israel; ^3^ Womens' Reproductive Mental Health research Unit, Psychiatric Department Tel Aviv Sourasky Medical Center Tel‐Aviv Israel; ^4^ Department of Counseling and Human Development University of Haifa Haifa Israel; ^5^ Lotem Center for Treatment of Sexual Trauma, Department of Psychiatry Tel Aviv Sourasky Medical Center Tel‐Aviv Israel; ^6^ Sackler Faculty of Medicine Tel‐Aviv University Tel‐Aviv Israel; ^7^ Sagol School of Neuroscience Tel‐Aviv University Tel‐Aviv Israel

**Keywords:** childhood abuse, fMRI, limbic activity, neuromodulation, PTSD

## Abstract

**Aim:**

Childhood sexual abuse (CSA) among women is an alarmingly prevalent traumatic experience that often leads to debilitating and treatment‐refractory posttraumatic stress disorder (PTSD), raising the need for novel adjunctive therapies. Neuroimaging investigations systematically report that amygdala hyperactivity is the most consistent and reliable neural abnormality in PTSD and following childhood abuse, raising the potential of implementing volitional neural modulation using neurofeedback (NF) aimed at down‐regulating amygdala activity. This study aimed to reliably probe limbic activity but overcome the limited applicability of functional magnetic resonance imaging (fMRI) NF by using a scalable electroencephalogram NF probe of amygdala‐related activity, termed amygdala electrical‐finger‐print (amyg‐EFP) in a randomized controlled trial.

**Method:**

Fifty‐five women with CSA‐PTSD who were in ongoing intensive trauma‐focused psychotherapy for a minimum of 1 year but still met *Diagnostic and Statistical Manual of Mental Disorders, Fifth Edition* (*DSM‐5*) PTSD criteria were randomized to either 10 add‐on sessions of amyg‐EFP‐NF training (test group) or continuing psychotherapy (control group). Participants were blindly assessed for PTSD symptoms before and after the NF training period, followed by self‐reported clinical follow‐up at 1, 3, and 6 months, as well as one session of amygdala real‐time fMRI‐NF before and after NF training period.

**Results:**

Participants in the test group compared with the control group demonstrated a marginally significant immediate reduction in PTSD symptoms, which progressively improved during the follow‐up period. In addition, successful neuromodulation during NF training was demonstrated.

**Conclusion:**

This feasibility study for patients with treatment‐resistant CSA‐PTSD indicates that amyg‐EFP‐NF is a viable and efficient intervention.

## Introduction

Child sexual abuse (CSA) is a global concern of epidemic proportion affecting children of all ages and racial, economic, and cultural backgrounds.^1^, ^2^, ^3^ Approximately one in five women reports a history of CSA in the Western World,^4^, ^5^ followed by protracted mental health outcomes and dysfunction.^6^ Chronic posttraumatic stress disorder (PTSD) is one of the most frequent psychopathological outcomes, affecting 38% to 51% of CSA survivors.^6^, ^7^, ^8^

CSA‐related PTSD (i.e. CSA‐PTSD) includes PTSD core diagnostic symptoms such as intrusive and distressing recurrent memories of the traumatic event, avoidance of trauma‐related reminders, negative emotional and cognitive changes, and hypervigilance.^9^ Critically, CSA‐PTSD responses to first‐line interventions (i.e. exposure‐based or psychopharmacology) are limited in efficacy and are associated with poor acceptance, compliance, and adherence rates,^10^, ^11^, ^12^ resulting in a particularly tenacious disorder.^13^, ^14^, ^15^ Moreover, across treatments, a significant proportion of PTSD cases (33%–60%) remain treatment refractory, posing an immense disease burden.^16^, ^17^, ^18^

One well‐identified neuropsychological dysfunction at the crux of CSA‐PTSD is emotion dysregulation,^19^ which has been extensively investigated from clinical^20^ and neural^21^ perspectives. Clinical emotion dysregulation is mostly well‐known for its hallmark deficits in dissociation tendencies,^22^, ^23^ whereas core neural emotion‐regulation dysfunctions have been strongly linked to amygdala hyperactivity both in PTSD^21^, ^24^ and specifically following childhood abuse.^25^

Accordingly, novel self‐neuromodulation procedures that target regulation of amygdala‐related activity may hold great therapeutic potential. One application of self‐neuromodulation procedures is through neurofeedback (NF), a method that involves volitional neural regulation guided by a closed‐loop brain‐computer interface procedure of reinforcement learning,^26^ which can be used to target specific dysfunctional processes by modifying underlying neural mechanism.^27^ Importantly for PTSD, in comparison to psychotherapy, this method does not necessarily require a painful return to traumatic memories, reducing emotional burden.

Intriguingly, healthy individuals have demonstrated successful down‐regulation of their amygdala activity in the presence of real‐time functional magnetic resonance imaging NF (rt‐fMRI‐NF), which was associated with emotion regulation enhancement.^28^ Expanding these findings to clinical populations, a current a meta‐analysis reported medium effect sizes of symptom reduction following limbic down‐regulation.^29^

While seemingly effective, implementing rt‐fMRI‐NF has a major scalability disadvantage (e.g. accessibility/mobility and affordability) that stands in its way to broadly being applied for clinical purposes in psychiatry.^30^ Electroencephalogram (EEG), on the other hand, has good accessibility and affordability but poor spatial resolution. To achieve treatment scalability while maintaining precise targeting of a specific, stable, and significant neural mechanism, we applied a validated NF approach of fMRI‐inspired EEG model of amygdala‐related activity termed amygdala electrical‐finger‐print (Amyg‐EFP).^31^, ^32^, ^33^, ^34^ This method uses machine learning on simultaneously recorded EEG and fMRI to generate a statistical model that enables signal transition between the two modalities.^33^, ^34^ This model has been previously validated in several independent samples by correlating simultaneously recorded transformed EEGs and right amygdala blood oxygenation level–dependent (BOLD) fMRI both in healthy^32^ and patient populations.^35^ Employing this method for the first time in chronic PTSD^36^ has intriguingly demonstrated promising initial evidence (i.e. learned limbic modulation and short‐ and long‐term clinical improvement).

Despite the critical need for more effective options in treatment‐resistant CSA‐PTSD and albeit emerging novel mechanism‐based approaches, to date, interventions that modulate limbic dysfunction (either standalone or adjunctive) have not been investigated among patients with CSA‐PTSD.

To address these shortcomings, we utilized amyg‐EFP‐NF for the first time in patients with CSA‐PTSD, conducting a 10‐session, randomized controlled adjunctive NF trial. Fifty‐five women who participated in ambulatory psychotherapy for a minimum of 1 year, and still met the *Diagnostic and Statistical Manual of Mental Disorders, Fifth Edition* (*DSM‐5*), criteria for PTSD (i.e. treatment‐resistant)^37^ were randomized between amyg‐EFP‐NF + psychotherapy or continuing psychotherapy. PTSD symptoms were blindly assessed before and after the NF training period, together with PTSD and emotion regulation dissociation self‐report measures, which were also collected 1, 3, and 6 months after NF. To verify and directly assess patients learned amygdala down‐regulation,^31^, ^32^ amygdala‐targeted fMRI‐NF was performed by both groups before and after the NF training period. The decision to compare NF add‐on therapy with psychotherapy alone was endorsed according to recent recommendations for treatment‐refractory PTSD, advising the addition of potent interventions with standard of care for enhanced therapeutic outcomes.^37^, ^38^ Second, we regard the current study as a feasibility trial among an extremely vulnerable clinical population, and thus maintaining current treatment was the ethically preferable option.

The study goals were to first investigate the clinical effect of adding amyg‐EFP‐NF training (test group) to psychotherapy alone (control group). We expected a greater reduction in PTSD symptoms immediately after NF treatment among patients in the test group compared with the control group. Based on prior amyg‐EFP‐NF findings in PTSD,^36^ we also hypothesized greater PTSD symptom reduction at long‐term follow‐up among patients in the test group compared with the control group. Our second goal was to assess the feasibility of repetitive amyg‐EFP‐NF regulation in the test group. We expected lower amyg‐EFP signal during the active regulation period relative to baseline. In addition, corresponding to previous investigations,^29^, ^39^ we expected more modulation in the last compared with the first NF sessions. Although this study was powered to find a clinical effect, in line with standard NF studies,^36^, ^40^ we exploratorily investigated whether the test group compared with the control group demonstrated greater amygdala BOLD down‐regulation in rt‐fMRI‐NF before to after the NF training period, and whether amyg‐EFP modulation correlated with clinical improvement.

## Method

### Participants and recruitment

Women who were treated in specialized outpatient clinics for survivors of CSA (all associated and supervised by a central clinic), who suffered from either single or multiple sexual abuse that occurred under the age of 18, participating in ongoing intensive trauma‐focused psychotherapy for a minimum of 1 year with insufficient responses^37^, ^41^ were offered participation in NF add‐on therapy. Study recruitment was advertised by posters and brochures located in clinics as well as by referrals from the clinics' psychiatrist. The psychotherapy treatment protocol relied on common and well‐established principles of interpersonal trauma treatment^42^, ^43^ and incorporated skills from dialectical behavioral therapy,^44^ which included psychoeducation, emotion regulation training, and trauma processing.^45^ Treatment included weekly individual psychotherapy, skills group, and psychotropic medication, widely accepted modules of CSA‐PTSD treatments.^46^ Assessment procedures were administered by certified psychologists or psychology graduate students supervised by certified psychologists and included PTSD evaluation (Clinician‐Administered PTSD Scale [CAPS‐5]) and a Structured Clinical Interview for DSM‐IV (SCID). Inclusion criterion included PTSD diagnosis according to CAPS‐5 and exclusion criteria included comorbidities associated with significant neural damage that may hinder the ability to benefit from treatment (i.e. major medical or neurological disorders, psychosis, schizophrenia and substance abuse).

Sample size was determined with a formal *a priori* power analysis using G*Power,^47^ applying the conventional power of 0.8, alpha of 0.05, and the weighted mean of clinical effect size in previous amyg‐EFP‐NF trials (η_p_^2^ = 0.16).^36^, ^39^ The analysis pointed to a required sample size of 45 participants to detect a reliable effect. Considering our expectation of an 80% retention rate,^13^ we recruited 55 participants.

### General Procedure

**Fig. 1:**
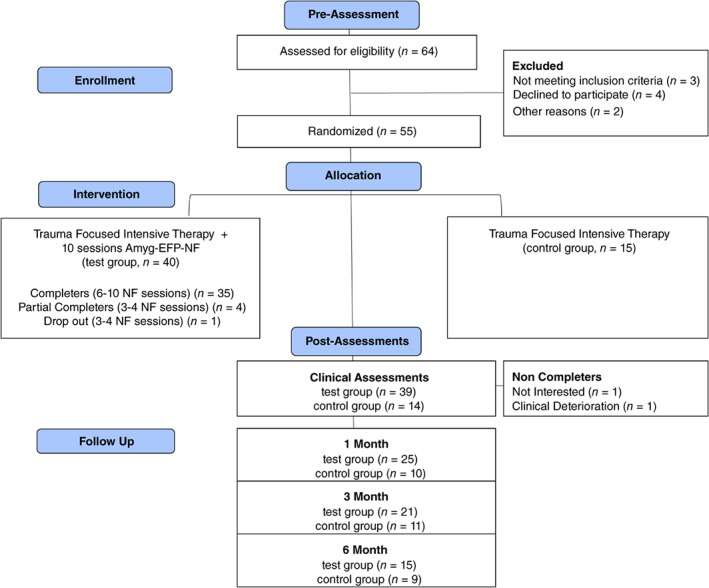
CONSORT (consolidated standards of reporting trials) diagram. Amyg‐EFP, amygdala electrical‐finger‐print; NF, neurofeedback.

**Fig. 2:**
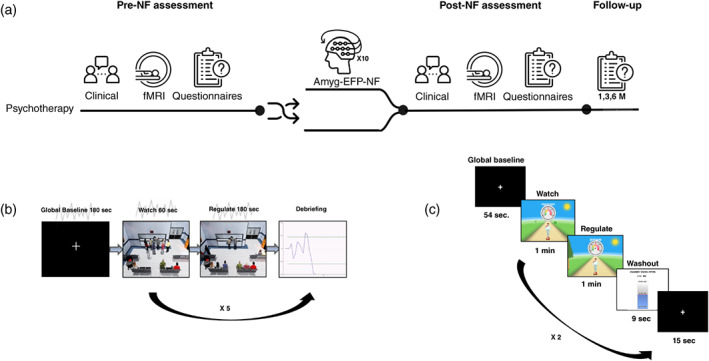
(a) Study design. Clinical assessments: functional magnetic resonance imaging neurofeedback (fMRI‐NF) scans were performed before (‘Pre‐NF’) and immediately after (‘Post‐NF’) the completion of NF. Following clinical assessment, patients were randomized either to continuing psychotherapy or adding NF training. Follow‐up self‐report assessments were performed at 1, 3, and 6 months following NF using online questionnaires: (b) amygdala electrical‐finger‐print (amyg‐EFP‐NF) training block; each session consisted of five repetitions of three consecutive conditions: ‘Watch’ (1 min), ‘Regulate’ (3 min), and debriefing with a graphic feedback on the signal modulation time‐course in addition to a 3 min ‘Resting‐State’ that appeared once in the beginning. During regulate, participants were instructed to down‐regulate the feedback stimuli by practicing self‐generated mental strategies. (c) Each rt‐fMRI‐NF session consisted of two cycles with three conditions: ‘Watch’ (60 s), ‘Regulate’ (60 s), and ‘Washout’ (bar feedback of the former NF block relative to its previous baseline block, 9 s, fixation, 15 s) in addition to a ‘Resting‐State’ (54 s) that appeared once in the beginning. During the ‘Regulate’ condition, amygdala BOLD activity relative to the ‘Watch’ condition (mean parameter estimates) was reflected in the skating speed displayed on a speedometer above the figure. Participants were instructed to decrease the riders' speed by practicing any mental strategy they see fit, and during the second session the amyg‐EFP‐NF group was encouraged to use techniques they found successful during the NF training phase.

Sixty‐four participants were assessed for eligibility, with 55 patients meeting CAPS‐5 criteria for PTSD (aged 21–65 years) enrolled and randomized in a blinded fashion balanced for age and CAPS‐5 score with 2.5/1 allocation^1^ (Fig. 1). All participants signed informed consent and anonymity was preserved. Amyg‐EFP‐NF training included 10 sessions. Primary outcome was based on CAPS‐5 administered before and immediately after NF sessions by clinicians who were blinded to treatment protocol. Participants underwent pre‐ and post‐assessment testing sessions of amygdala‐targeted rt‐fMRI‐NF. Follow‐up measures were collected at 1, 3, and 6 months after completion of NF training and included self‐report PTSD (PTSD Checklist for *DSM‐5* [PCL‐5]) and dissociation measures (Dissociative Experiences Scale [DES‐II]) (Fig. 2a). All participants were guided to maintain their treatment plan during the study and received monetary compensation.

### NF procedures

#### Amyg‐EFP‐NF training protocol

The amyg‐EFP‐NF training protocol included 10 sessions, administered twice a week for 2 weeks and then weekly. Each session (Fig. 2b) lasted ~50 min including EEG preparation time and began with a 3‐min ‘resting‐state’ recording using the research‐grade EEG device. Following baseline, participants were trained to down‐regulate their amyg‐EFP by receiving feedback on their success *via* a three‐dimensional audiovisual animated scenario that has been shown to induce higher engagement and a more sustainable learning effect than a simple unimodal two‐dimensional interface.^48^ Each NF cycle consisted of two consecutive conditions, repeated five times: active baseline, passively watching the interface (Watch; 60 s), down‐regulating amyg‐EFP (Regulate; 180 s), followed by a debriefing graph that presented regulation success and a set of questions regarding the mental strategies employed. During ‘Watch,’ participants were guided not to consider any mental strategies or previous successes or failures, stressing the importance of creating a significant mental difference between conditions. During ‘Regulate,’ a free and uninstructed exploration of mental strategies was encouraged in order to allow participants to adopt individual strategies that are most effective. The feedback interface consisted of a three‐dimensional audiovisual scenario including avatars and sound of chatter and commotion in a busy emergency room. With lower amyg‐EFP relative to baseline (measured every 3 s), the avatars' position incrementally changes from standing to sitting with a matching soundtrack of a noisy emergency room complementing the system output. During both conditions, 75% of the characters congregate at the front desk, expressing agitation through body movements and verbal sounds. While during the ‘Watch’ condition, this arousal level remained constant, during the ‘Regulate’ condition it changes according to the amyg‐EFP signal. The system was implemented using the Unreal Development Kit game engine (Epic Games, Inc.), which controls relevant animations (sitting, standing, and protesting), as well as their transitions for individual characters (*cf* ^37^, ^38^, ^40^).

#### EEG acquisition and online calculation

EEG data were acquired using the V‐Amp EEG amplifier (Brain Products) and the BrainCap electrode cap with sintered Ag/AgCI ring electrodes (Falk Minow Services). Electrodes were positioned according to the standard 10/20 system. The reference electrode was between Fz and Cz. The raw EEG signal was sampled at 250 Hz and recorded using the Brain Vision Recorder software (Brain Products). Amyg‐EFP amplitude was calculated based on data recorded from the Pz channel.^33^, ^34^

##### NF online calculation

Online EEG processing was conducted *via* RecView software (Brain Products), which was custom modified to enable export of the corrected EEG data in real‐time through a TCP/IP socket. Amyg‐EFP signal was calculated online using a statistical model previously developed to enable the prediction of localized activity in the amygdala BOLD from a time‐frequency decomposition of the EEG.^34^ This was originally accomplished by applying machine learning algorithms on EEG data acquired simultaneously with fMRI and validated on an external data set.^32^ In short, a 12‐s long raw EEG segment is notch‐filtered for 50Hz line interference, and then converted to a time‐frequency representation using the S‐transform.^49^ The signal is down‐sampled to 4 Hz and the frequency domain is reduced to 10 frequency bands defined using an energy uniformity constraint on the EEG data. Each resulting time‐frequency bin is then multiplied by a predefined weight and summed to a final estimate of the current fMRI activity.^33^ Preprocessing algorithm and signal calculation models were compiled from MATLAB R2009b (The MathWorks, Inc.) to Microsoft.NET in order to be executed within the Brain Vision RecView EEG Recorder system. Data were then transferred to a MATLAB.NET‐compiled DLL that calculated the value of the targeted signal power every 3 s. During each ‘Regulate’ condition, an amyg‐EFP sample was generated every 3 s and normalized to the distribution of the EFP samples acquired during the preceding ‘Watch’ condition according to the following formula:

$$Z_{\mathrm{EFP}}=\frac{{\mathrm{EFP}}_{\text{Regulate}}-\mu_{\text{Watch}}}{\sigma_{\text{Watch}}}$$

where $\mu_{\text{Watch}}$ and $\sigma_{\text{Watch}}$ are the average and standard deviation of the ‘Watch’ condition samples, respectively. This score was then transformed to a scale of 1 to 10 and transferred to the animated scenario to generate the audiovisual feedback.

##### Offline EFP calculation

Regulation success was computed in each block by subtracting ‘Watch’ (active baseline) from ‘Regulate,’ divided by the SD of ‘Watch.’ Success index is a continuous measure ranging from positive to negative (down‐regulation).

#### rt‐fMRI NF procedure

Patients without MRI‐excluding criteria completed one assessment session of amygdala‐fMRI‐NF before and after the amyg‐EFP‐NF training period, each consisting of two consecutive cycles. To test NF skill transferability between contexts and to refute the possibility that observed group differences are merely a result of familiarity with the animated scenario, the fMRI‐NF was of a similar block design as in the EFP‐NF procedure but with a different, two‐dimensional unimodal graphic interface with an animated figure standing on a skateboard skating down a rural road (Fig. 2c). In addition to the ‘Watch’ (60 s) and ‘Regulate’ (60 s) conditions, a ‘Washout’ condition was added, during which participants were instructed to cease self‐reflecting and were informed *via* graphics of their average speed representing their success in the former NF block relative to its previous baseline block (9 s), followed by a fixation interval (15 s). During the ‘Regulate’ condition, amygdala BOLD activity relative to the ‘Watch’ condition (mean parameter estimates) was reflected in the skating speed ranging between 50 and 130 km h^−1^ displayed on a speedometer above the figure. Participants were instructed to decrease the riders' speed by practicing any mental strategy they see fit, and during the second session the EFP‐NF group was encouraged to use techniques they found successful during NF training.

##### fMRI Offline Analysis

Preprocessed BOLD data from the before and after scans were used in two separate general linear models performed with SPM12. Each model included three regressors for the experimental conditions (‘Watch,’ ‘Regulate,’ ‘Washout’). Regressors were convolved with a canonical hemodynamic response function. Additional nuisance regressors included the six head‐movement realignment parameters, their temporal derivatives and quadratic terms, and a single regressor for each motion outlier detected. For each subject and time point, a single contrast map of ‘Watch‐Regulate’ was generated, and an averaged value of the right amygdala region of interest was extracted for further analysis.

See supplementary materials (Appendix A) for fMRI data acquisition, online feedback calculation, and fMRI offline preprocessing.

### Outcome measures

The following interviews and questionnaires were used for assessment.

CAPS‐5—the gold‐standard structured clinician interview for assessing PTSD diagnosis and symptom severity.^50^, ^51^ CAPS contains explicit, behaviorally anchored probes for each of the 20 symptoms of the *DSM‐5* (on a scale of 0–4) that are summed to a total score.

PCL‐5—A 20‐item self‐administered inventory that indexes PTSD symptoms in the past month, rated on a scale of 0 to 4 and summed to a total score.^52^

DES‐II—A 28‐item self‐administered measure of frequency of dissociative experiences build on the assumption of a “dissociative continuum,” rated on a scale between 0 and 100 and summed to a total score.^53^, ^54^

### Data Analysis

SPSS version 23 (IBM) was used for statistical analysis. To evaluate treatment‐immediate effect, we used ancova, controlling for pre‐CAPS‐5 scores with between‐subject factor of group, and post–CAPS‐5 scores as the dependent variable. For clinical efficacy assessment, we calculated the number needed to treat based on achieving loss of PTSD diagnosis (according to CAPS‐5) representing the number of patients who needed to be treated with amyg‐EFP‐NF add‐on for one patient to lose diagnosis compared with the group without add‐on.^55^ To evaluate the treatment's long‐term effect, we used two complementary analyses: first, we used intention‐to‐treat analysis, which included all randomized patients, and applied linear mixed‐model analysis, which is fit for multiple measurements, and incomplete data with a between‐subject fixed group factor (2) covariate for time point (5) and clinical self‐report scores as the dependent variable. Missing data were handled using restricted maximum‐likelihood estimation, and a compound symmetry covariance structure was assumed. Second, we employed a per‐protocol control analysis of including only completers of the full study. To evaluate NF training modulation, we employed a repeated‐measures anova with condition (‘Watch’ and ‘Regulate’) and session number as a within‐subject factor, and amyg‐EFP signal as the dependent variable. All reported *P*‐values are two tailed, and we provide model fit estimates that include partial eta‐squared and *F* values.

## Results

The Table 1 shows that there were no significant pre‐treatment group differences in demographic and psychopathological characteristics.

**Table 1:** Demographic and psychopathological characteristics by group before treatment

			Statistics	
	Test group (*n* = 40), mean (SD)	Control group (*n* = 15), mean (SD)	*t*	*P‐*value
Demographics				
Age (SD)	37.37 (11.45)	35.86 (9.43)	0.45	0.65
Years of education (SD)	14.23 (3.31)	14.46 (4.36)	−2.01	0.84
Clinical characteristics				
Time since trauma	28.91 (12.28)	28.36 (8.67)	0.15	0.87
CAPS‐5 (SD)	40.52 (9.92)	43.06 (10)	−0.84	0.4
PCL‐5 (SD)	42.97 (16.83)	44.86 (19.05)	−0.35	0.72
DES‐II (SD)	28.93 (19.06)	32.33 (21.51)	0.56	0.57

Abbreviations: CAPS‐5, Clinician‐Administered PTSD Scale; DES‐II, Dissociative Experiences Scale; PCL‐5, PTSD Checklist for *DSM‐5*.

### Clinical effects

High adherence rates were observed both to the EFP‐NF protocol (88%, six of 10 NF session completers [*cf*,^36^, ^39^ reported a minimum of six sessions is needed to acquire a meaningful capacity of regulation]) and to the study protocol (92%, including participants who completed clinical evaluation after treatment regardless of NF adherence). The follow‐up measurement response rate was similar between study groups and showed moderate adherence rates.

**Fig. 3:**
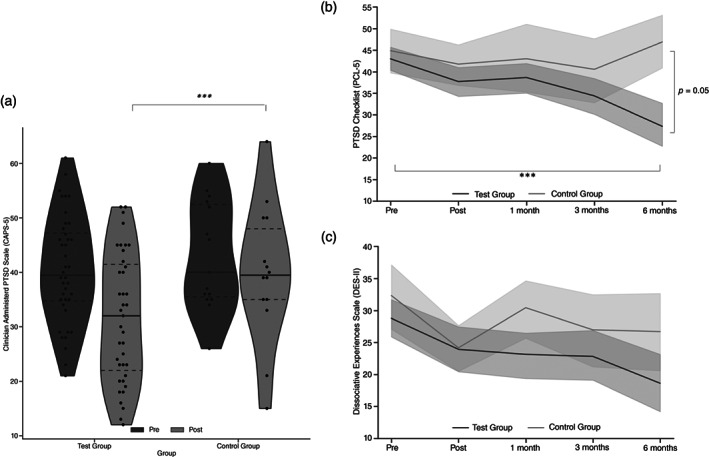
(a) Immediate clinical effect. Total Clinician‐Administered PTSD Scale (CAPS‐5) score (y‐axis) reflecting the severity of posttraumatic stress disorder (PTSD) symptoms before (“Pre”) and after (“Post”) assessments for the test and control groups. The dashed line represents the first and third quartiles; the solid line represents the mean. Results demonstrate a marginally significant reduction of CAPS‐5 score following the add‐on in the test group compared with the control group. (b) Full‐term clinical effect. Total PTSD Checklist for *DSM‐5* (PCL‐5) scores (y‐axis) before, after, and at 1, 3, and 6 months (x‐axis) showing a pronounced decrease in self‐report PTSD severity in the test group compared with the control group. Post hoc analyses revealed a marginally significant PCL‐5 reduction between groups at 6 months and a significant effect between pre‐neurofeedback and 6‐month follow‐up. (c) Full‐term emotion regulation effect. Total Dissociative Experiences Scale (DES‐II) scores (y‐axis) before, after, and at 1, 3, and 6 months (x‐axis) showing a pronounced decrease in emotion regulation dissociation tendencies in the test group compared with the control group. **P ≤ 0.01, ***P ≤ 0.001.

In order to investigate immediate clinical effectivity, a Levene test and normality checks were performed and demonstrated that the assumptions were met (confirming that unbalanced group size did not violate assumptions). One participant from each group was not included in this analysis due to missing data at the post‐intervention timepoint. In accordance with our hypothesis, we found a marginally significant improvement in PTSD symptoms in the test group compared with the control group (Fig. 3a). One‐way ancova revealed a marginally significant reduction in CAPS‐5 total scores (*F*_(1,52)_=3.88 [*P* = 0.054], *η*_p_^2^ = 0.07) (test group: *M* = 31.66 [SD = 11.83]; control group: *M* = 39.78 [SD = 12.51]), exemplified by a numeric improvement in the test group of almost three times the size of the control group (i.e. symptom reduction of 8.86 and 3.28 points, respectively). Furthermore, CAPS‐5 symptom reduction percentage pre‐ to post‐NF marginally differed between groups (*t* (51)=1.778, *P* = 0.08). We secondarily investigated CAPS‐5 subscales, which revealed that PTSD reduction was global and not driven by a specific symptom cluster (one‐way ancova: re‐experiencing [*F*_(1,52)_=2.09, *p*FDR = 0.19], avoidance [*F*_(1,52)_=3.88, *p*FDR = 0.21], alterations in mood and cognition [*F*_(1,52)_=1.86, *p*FDR = 0.17], and arousal [*F*_(1,52)_=2.32, *p*FDR = 0.26]).

Group comparison following NF add‐on therapy showed a number‐needed‐to‐treat of 4.04 (absolute risk reduction, 24.7% [95% confidence interval (CI), 1.86%–51.31%]) replicating prior findings^36^ and indicating that 4.04 persons were needed to get the favorable outcome (i.e. loss of PTSD diagnosis) compared with the control group, expressing the magnitude of add‐on amyg‐EFP‐NF treatment over standard care alone.

Confirming our hypothesis, long‐term PTSD symptoms (PCL‐5 scores) significantly decreased among the test group compared with the control group (Fig. 3b, intent‐to‐treat linear mixed‐model analysis (including all randomized participants)) demonstrated by a significant time‐by‐group interaction (*F*_(1,84.28)_ = 5.59, *P* = 0.01 [95% CI, −4.81 to −0.43]): time (*F*_(1,139.69)_ = 10.04, *P* = 0.01) and group (*F*_(1,84.28)_ = 0.02, *P* = 0.87). Complementary per‐protocol (completers cohort) repeated‐measures anova analysis demonstrated a similar significant time‐by‐group interaction (*F*_(1,68)_=3.76, *P* = 0.008, *η*_p_^2^ = 0.18): time and group (*F*_(1,14)_=1.45, *P* = 0.22, *F*_(1,14)_=1.63, *P* = 0.21, respectively; see attrition bias analysis in Supplementary Material Appendix B). Planned post hoc analyses between groups revealed a nearly significant PTSD symptom reduction at 6 months with greater improvement in the test group compared with the control group (*t*(25)= − 2.06, *P* = 0.05) (test group: *M* = 27.47 [SD = 18.4]; control group: *M* = 42.9 [SD = 22.5]). Finally, planned post hoc within‐test group findings revealed a significant improvement between pre‐NF and 6‐month follow‐up and marginal improvement with 3‐month follow‐up (pre‐NF: *M* = 39.93 [SD = 18.64]; 6 months: *M* = 27.75 [SD = 18.21], *t*(15)=4.28, *P* = 0.001; 3 months: *M* = 36.15 [SD = 19.07], *t*(18)=1.99, *P* = 0.06).

An exploratory analysis revealed that long‐term emotion dysregulation dissociation tendencies (DES‐II scores) significantly decreased among the test group compared with the control group (See Fig. 3c, intent‐to‐treat linear mixed‐model analysis [including all randomized participants]) demonstrated by a time‐by‐group interaction trend (*F*_(1,138.66)_ = 2.08, *P* = 0.1 [95% CI, −3.91 to 0.6]): time (*F*_(1,138.66)_ = 8.81, *P* = 0.004) and group (*F*_(1,91.55)_ = 0.006, *P* = 0.94). However, complementary per‐protocol (completers cohort) repeated‐measures anova analysis demonstrated a significant time‐by‐group interaction (*F*_(1,64)_=3.45, *P* = 0.037, *η*_p_^2^ = 0.17): time (*F*_(1,13)_=2.83, *P* = 0.06) and group (*F*_(1,13)_=0.09, *P* = 0.76). Post hoc analyses did not reveal any significant comparisons.

### Amyg‐EFP signal modulation during training

**Fig. 4:**
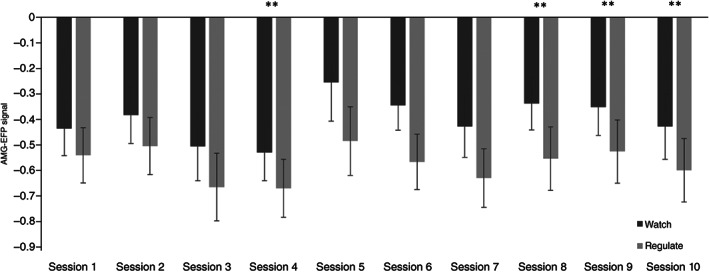
The black bar denotes the ‘Watch’ condition and the gray bar denotes the ‘Regulate’ condition with standard error bars of each condition. The x‐axis represents session number, and the y‐axis represents the mean across amygdala electrical‐finger‐print (Amg‐EFP).

According to our hypothesis, we found successful EFP neuromodulation demonstrated by more modulation in the‘Regulate’ compared with the ‘Watch’ condition and a significate interaction between session and condition, indicating that session progression involved more modulation (See Fig. 4, repeated‐measures anova analysis, condition (*F*_(1,20)_=57.06, *P* < 0.0001, *η*_p_^2^ = 0.66, ‘Regulate’ *M* = −0.58 [SD = 0.06]; ‘Watch’ *M* = −0.4 [SD = 0.06], condition by session (*F*_(1,9)_ = 1.96, *P* = 0.044, *η*_p_^2^ = 0.06) session (*F*_(1,9)_=0.41, *P* = 0.92). Quantifying learning effect by calculating the difference between the average of the first five NF sessions compared with the last five sessions revealed a significant effect demonstrating enhanced regulation in the last versus the first sessions (*t*(34) = 2.06, *P* = 0.046; first: *M* = −0.58 [SD = 0.46]; last: *M* = −0.82 [SD = 0.85]).

### Target engagement indications

**Fig. 5:**
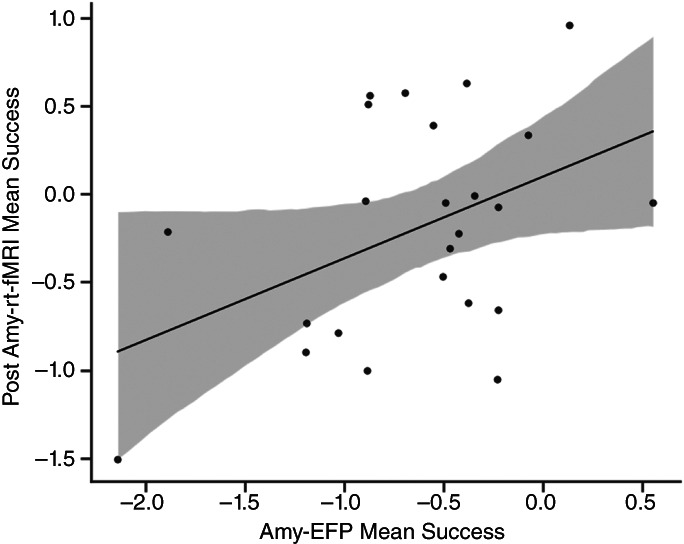
The neurofeedback (NF) learning effect correlation demonstrating target engagement and transferability between NF contexts; The x‐axis represents the average modulation across amygdala electrical‐finger‐print (amyg‐EFP) sessions and the y‐axis represents the average amygdala modulation after real‐time functional magnetic resonance imaging (rt‐fMRI) session.

An exploratory analysis validated amyg‐EFP learning with rt‐fMRI‐NF modulation and demonstrated transferability between contexts in the test group; greater down‐regulation of amyg‐EFP signal during NF training (average modulation across sessions) was significantly correlated with greater down‐regulation of amygdala BOLD activation during post‐training rt‐fMRI scanning (average modulation) (*r*(24)=0.43 *P* < 0.05) (Fig. 5).

Last, exploratory differences between amygdala fMRI‐NF modulation pre‐ to post‐intervention were found only at the descriptive level and did not yield significant differences (ancova, controlling for amygdala BOLD down‐regulation in rt‐fMRI pre‐NF training with a between‐subject factor of group [*F*_(1,27)_=0.46, *P* = 0.50, test group: *M* = −0.19 (SD = 0.63); control group: *M* = 0.11 (SD = 0.6)]) (for further analysis see Supplementary Material Appendix B). Finally, in an exploratory and underpowered analysis, no significant association was found between amyg‐EFP signal modulation and clinical change (reflected by delta pre‐ to post‐total CAPS‐5 scores) (*r*(38)=0.1 *P* = 0.26).

## Discussion

To our knowledge, this is the first study to examine a scalable amygdala‐related EEG‐NF add‐on intervention for patients with treatment‐resistant CSA‐related PTSD. As hypothesized, NF add‐on to psychotherapy resulted in a marginaly significant immediate PTSD symptom reduction (i.e. CAPS‐5 score) relative to psychotherapy alone (Fig. 3a). This clinical effect was enhanced in follow‐up self‐assessments at 3 and 6 months, as depicted by the PCL‐5 (Fig. 3b) and DES‐II scores (Fig. 3c) showing larger reductions in the test group compared with the control group. Second, as expected, patients who exercised NF demonstrated successful down‐regulation of the amyg‐EFP signal in ‘Watch’ versus ‘Regulate’ conditions (Fig. 4). Third, in an exploratory analysis, the engagement of the amygdala in NF training was validated by a strong association between down‐regulation in rt‐fMRI‐NF with greater down‐regulation of amyg‐EFP during NF training (Fig. 5). Last, this study was not powered and did not find pre‐ to post‐NF rt‐fMRI‐NF amygdala down‐regulation differences between groups, nor did amyg‐EFP modulation correlate to clinical improvement.

### Clinical effect

A critical concern in developing novel intervention protocols in PTSD,^56^ and especially in treatment‐resistant CSA populations, regards protocol adherence and compliance.^13^ The current investigation showed nonstratified high rates of adherence both to the protocol and to clinical assessments (88% and 92%, respectively) exceeding other NF protocols (79%)^57^ and evidence‐based psychological treatments in childhood abuse PTSD (75%),^17^ and medication adherence in mixed trauma PTSD (76%).^58^ These findings might partially stem from the unprovocative nature of the implemented NF protocol, avoiding symptom exacerbation that can contribute to high dropout rates,^59^, ^60^ as well as adjunctive psychotherapy that provided ongoing support.

Clinical improvement in the current investigation revealed a medium effect size in the amyg‐EFP‐NF add‐on group compared with psychotherapy alone,^61^ presented by a global reduction in PTSD symptoms. This effect is comparable with standard interventions in childhood‐trauma PTSD^18^ but could be considered superior since it was conducted among a treatment‐refractory population. In addition, the test group showed an 8.86‐point CAPS‐5 total score reduction compared with 3.28 in the control group, which is considered within the meaningful range of change.^57^, ^62^, ^63^

Clinical improvement significantly intensified during follow‐up measurements, showing that the test group showed further improvement in the months after NF completion compared with the control group. The temporal pattern of symptom alleviation following NF is an emerging characteristic following NF training^36^, ^39^, ^64^ and might be explained by the possibility of long‐term brain function alteration^65^, ^66^, ^67^ involving a series of consolidation and reconsolidation processes.^68^, ^69^ Some evidence supports this premise showing that NF alters the correlational structure of network brain activity after the intervention^65^, ^66^, ^70^, ^71^, ^72^, ^73^ which, following Hebbian principles,^74^, ^75^ may be self‐reinforced and therefore increasingly coupled over time. Such mechanistic speculations have been previously discussed^76^ and should be further investigated.

### Learned neuromodulation

Corresponding to previous EFP findings,^36^, ^39^ patients learned to down‐regulate amyg‐EFP signals in an emotionally agitated interface, thus reinforcing that individuals with severe psychiatric disorders can volitionally modulate their neural functions. In order to reduce unknown variance and adhere to formed and well‐established NF parameters, this protocol relied on a previously established and efficient interface^29^, ^48^ and implemented the average add‐on number of sessions reported in adjunctive PTSD trials.^38^ In general, NF learning dynamic variations^77^, ^78^, ^79^ could be influenced by several factors,^80^ which should be further tested in a general matter and specifically per disorder, in order to facilitate maximum learning while specifying a dose‐effect relationship in multisession NF.^27^

The current study validated self‐regulation of amyg‐EFP with rt‐fMRI‐NF activity, demonstrating target engagement and transferability between contexts. This corresponds to previous findings showing that the amyg‐EFP correlated with simultaneously acquired right amygdala BOLD activity^32^ and reports that amyg‐EFP‐NF training (relative to sham or no‐NF control) resulted in better amygdala self‐regulation as measured by rt‐fMRI‐NF.^31^, ^32^, ^36^ It is important to note that this study was not designed and did not find neural changes in amygdala rt‐fMRI‐NF following training nor brain‐behavior correlations similar to previous amyg‐EFP‐NF investigations in PTSD.^36^ This is concurrent with the notion that brain alterations are ongoing^64^ and therefore neural markers may reveal differences over time. In addition, this finding is concurrent with recent reviews that suggest larger samples are needed to infer such changes and whole‐brain signatures in order to exploit the high dimensionality inherent in fMRI data.^81^, ^82^

### Future directions and limitations

This study constitutes a randomized controlled feasibility trial, and, as such, it has demonstrated that patients with treatment‐resistant CSA‐PTSD are able to modulate their neural functions, adhere to a protocol, and gain short‐ and long‐term clinical benefit. Despite the novelty of the study, it is important to mention several limitations and future directions.

First, a carefully randomized, controlled, double‐blind trial is required to more precisely evaluate the contribution of actual amyg‐EFP modulation over and above unspecific NF effects including placebo. Specifically, designing adequate control arms in NF studies is a challenge that recently gained attention due to difficulty in accounting for NF confounders.^27^ Methodological considerations include meticulous attention to concurrent modulation of other processes that are *not* operated in the experimental intervention as well as modulation of NF‐general processes (i.e. control, reward, and learning processes), which are essentially different from the experimental intervention. For example, using yoked‐sham NF induces a lack of contingency between neural patterns and the feedback, which might lead to major differences in NF reward processes. That is, participants may deduce that they are not receiving veritable feedback and thus may reduce their motivation, task engagement, and positive expectations in comparison to a genuine feedback group.^83^ Moreover, even when matching feedback variability between groups by ‘yoking’ in a double‐blinded manner, there would still exist differences in NF learning, as no learning based on contingencies between feedback and neural patterns would occur. This matter has significant ethical implications in the context of implementing an NF sham‐control in the treatment of vulnerable nonresponders with CSA‐PTSD. Nevertheless, large‐scale NF studies with more control conditions are needed to establish NF as a treatment of choice in psychiatry.

Second, we established an important direct association between amyg‐EFP regulation and amygdala recruitment in rt‐fMRI‐NF following intervention. This is particularly important since we cannot rule out indirect involvement of other brain regions associated with the amyg‐EFP fingerprint. As shown in Keynan et al,^32^ amyg‐EFP–correlated BOLD activations extend beyond the right amygdala to lateral temporal and occipital regions, which are in closer proximity to the scalp. Thus, the underlying neural mechanism of the observed effect warrants further research in larger sample sizes and investigations of whole‐brain neural changes following treatment. Furthermore, the scalable nature of the amyg‐EFP, providing a prediction of deep brain activity from a single posterior scalp channel, exposes the recorded signal to transient sensory inputs from the NF interface, affecting the predicted signal. This may have been avoided by developing a more complex fingerprint, but our choice of an active baseline period (‘Watch’) for calculating the online regulation effect minimizes these interferences by matching the auditory and visual outputs from the environment between conditions. In addition, in add‐on designs, further efforts should aim to tease apart mechanisms of change specific to the NF intervention relative to psychotherapy^84^ and, accordingly, refine therapeutic protocol.^38^

Third, in this study, we assessed PTSD with the gold‐standard CAPS‐5, referring to the sample as PTSD following CSA as widely accepted in the literature.^13^ Future studies should also investigate the recently proposed self‐report measures of complex PTSD.^85^ Last, while there is accumulating evidence and recommendations towards sex‐specific interventions accounting for differential underlying mechanisms,^86^, ^87^ we suggest that this intervention also be investigated in a cohort of men.

## Author contributions

MB, TH, ZS, and LH were involved in the conception and design of the study; and NF, LH, GG, and DA were involved in the acquisition and analysis of data. All authors contributed to drafting the manuscript.

## Financial support

This work was supported by the Israel Science Foundation (MB, grant No. 2107/17; TH grant No. 2923/20), European Union's Horizon 2020 Framework Programme for Research and Innovation (grant No. 945539, Human Brain Project SGA3), The Innovation Authority – Kamin Program, National Institute of Psychobiology for Israel Young Investigator (LH); and the Brain and Behavior Foundation, NARSAD (LH, grant No. 26302).

## Disclosure statement

Talma Hendler is the Chief Medical Scientist and Chair of advisory board in GrayMatters Health Co Haifa Israel, and has stock options. This company sells the platform of “Prism for PTSD”, neufeedback driven by amygdala‐related biomarker, which is also used as the NF probe in this study. Other authors declare no conflict of interest.

## Ethics Statement

The authors assert that all procedures contributing to this work comply with the ethical standards of the relevant national and institutional committees on human experimentation and with the Declaration of Helsinki 1975, as revised in 2008. All procedures involving human subjects/patients were approved by Tel‐Aviv Sourasky Medical Center's institutional review board and registered at ClinicalTrials.gov (NCT03416764).

## Supporting information


**Appendix A.** fMRI Data acquisition and online feedback calculation.
**Appendix B.** Supplementary analyses.

## Footnotes

^1^
This study was designed to first compare NF augmentation to control group and secondly, to investigate an additional subdivision of groups (NCT03416764). Therefore, unbalanced group randomization was chosen in order to ensure that a sufficient number of participants adhere to the NF protocol, and additionally enables sufficient allocation to the preregistered subsamples (i.e. differing on estrogen levels).
