# Supplementary material for: Amygdala‐related electroencephalogram neurofeedback as add‐on therapy for treatment‐resistant childhood sexual abuse posttraumatic stress disorder: feasibility study
Source: Psychiatry Clin Neurosci. 2023 Dec 22;78(1):19–28. doi: 10.1111/pcn.13591 (PMC11488636; doi:10.1111/pcn.13591)
Supplement: Supplementary file 1 — Appendix A. fMRI Data acquisition and online feedback calculation. Appendix B. Supplementary analyses. [file PCN-78-19-s001.docx]

**Supplementary Materials**

**Appendix A**

**fMRI Data Acquisition and Online Feedback Calculation:** Structural and functional MRI scans were performed in a 3.0T Siemens MRI system (MAGNETOM Prisma) using a 20-channel head coil. To allow high-resolution structural images, a T1-weighted three-dimensional (3D) sagittal MPRAGE pulse sequence (repetition time/echo time = 1,860/2.74 ms, flip angle = 8°, pixel size = 1 × 1 mm, field of view = 256 × 256 mm) was used. Functional whole-brain scans were performed in an interleaved top-to-bottom order, using a T2*-weighted gradient echo planar imaging pulse sequence (repetition time/echo time = 2,500/30 ms, flip angle = 82°, pixel size = 2.3 mm, field of view = 220 × 220 mm, slice thickness = 3 mm, 42 slices per volume).

During the fMRI-NF sessions, activity from the right Amygdala was delivered as feedback to the participants. The probing of amygdala-BOLD for NF was based on a 6-mm sphere in Talairach space in the right amygdala (coordinates, 20, -5, -14) in correspondence to the Amygdala BOLD used as a predictor for the AmygEFP model (40). Momentary beta weights of the predefined ROI (averaged across all voxels of the ROI) were extracted online using Turbo Brainvoyager 3.0 (Brain Innovation). The beta weights were then transferred to MATLAB^TM^. For each TR, right amygdala activity was first converted to standardized score:

$$zBold\left( t \right)=\frac{B\left( t \right)- \mu\left( B_{BL} \right)}{\sigma\left( B_{BL} \right)}$$

Where B(t) is the right amygdala BOLD activity value at time point *t*, $\mu\left( B_{BL} \right)$ is the mean BOLD value during the Previous 'Watch' block. Values varied from -4 std. below baseline average to 4 std. above average. Next, each value was transferred into a speed scale using the following formula:

$$Speed\left( t \right)=60+15*zBold\left( t \right)$$

This equation results in the fact that the right amygdala BOLD value that equals the Previous 'Watch' average value, is set to 60km/h and each increase or decrease of 1 std. results in a 15km/h change. The -4 to 4 limit translates to a possible speed range of 0-120km/h. Moreover, in order to make the NF paradigm more interactive, flexible for modulation and challenging, an updating principle was incorporated. During 'Regulate' block, represented values were limited by an interval of 4 std., resulting in a 60km/h interval in speed scale. In the first block of the first run, the presented interval was symmetric around the average speed (60km/h): -2 to 2 (30km/h to 90km/h). In each consecutive run, the interval was updated based on the patients' success in the previous run, according to the following steps:

1. During the first cycle the interval in the NF screen is set to -2 to 2.
2. When the NF screen terminates a success index of the current block is calculated thus (n indicating the cycle's ordinal number):

$$Success_{Index\left( n \right)}=\frac{\mu\left( B_{NF\left( n \right)} \right)}{pooled\left( \sigma\left( B_{BL\left( n \right)} \right)+ \sigma\left( B_{NF\left( n \right)} \right) \right)}$$

By dividing the mean NF standardized value with the pooled average, we introduce the index not only with the subject's ability to successfully regulate his or her right amygdala activity during the 'Regulate' block, but also his or her ability to maintain it as constant as possible during the 'Watch' block.

1. The interval [lower limit upper limit] for the next cycle (n+1) is updated to be [Lower lim = (success_index(n) - learning_rate) Upper lim = (success_index(n) - learning_rate + 4)] The learning rate parameter is preset to: learning rate = 1.

This process is aimed at enabling significant regulation in one run to result in further regulation in the following run. This makes the NF paradigm more challenging and dynamic and pushes towards maximizing regulation.

**fMRI Offline Preprocessing and Analysis:** Preprocessing was done using *fMRIPrep* version 20.0.2 which is based on *Nipype* 1.4.2. The T1-weighted (T1w) image was corrected for intensity non-uniformity (INU) with 'N4BiasFieldCorrection` [@n4], distributed with ANTs 2.2.0 [@ants, RRID:SCR_004757], and used as T1w-reference throughout the workflow. The T1w-reference was then skull-stripped with a *Nipype* implementation of the `antsBrainExtraction.sh` workflow (from ANTs), using OASIS30ANTs as target template. Brain tissue segmentation of cerebrospinal fluid (CSF), white-matter (WM) and gray-matter (GM) was performed on the brain-extracted T1w using `fast` [FSL 5.0.9, RRID:SCR_002823,]. Volume-based spatial normalization to one standard space (MNI152NLin2009cAsym) was performed through nonlinear registration with `antsRegistration` (ANTs 2.2.0), using brain-extracted versions of both T1w reference and the T1w template. The following template was selected for spatial normalization: *ICBM 152 Nonlinear Asymmetrical template version 2009c*. For each of the functional runs the following preprocessing was performed. First, a reference volume and its skull-stripped version were generated using a custom methodology of *fMRIPrep*. The BOLD reference was then co-registered to the T1w reference using `flirt` [FSL 5.0.9, @flirt] with the boundary-based registration [@bbr] cost-function. Co-registration was configured with nine degrees of freedom to account for distortions remaining in the BOLD reference. Head-motion parameters with respect to the BOLD reference (transformation matrices, and six corresponding rotation and translation parameters) are estimated before any spatiotemporal filtering using `mcflirt` [FSL 5.0.9, @mcflirt]. BOLD runs were slice-time corrected using `3dTshift` from AFNI 20160207 [@afni, RRID:SCR_005927]. The BOLD time-series (including slice-timing correction when applied) were resampled onto their original, native space by applying the transforms to correct for head-motion. These resampled BOLD time-series will be referred to as *preprocessed BOLD in original space*, or just *preprocessed BOLD*. The BOLD time-series were resampled into standard space, generating a *preprocessed BOLD run in MNI152NLin2009cAsym space*. First, a reference volume and its skull-stripped version were generated using a custom methodology of *fMRIPrep*. Several confounding time-series were calculated based on the *preprocessed BOLD*: framewise displacement (FD), DVARS and three region-wise global signals. FD and DVARS are calculated for each functional run, both using their implementations in *Nipype* [following the definitions by @power_fd_dvars]. The three global signals are extracted within the CSF, the WM, and the whole-brain masks. The head-motion estimates calculated in the correction step were also placed within the corresponding confounds file. The confound time series derived from head motion estimates and global signals were expanded with the inclusion of temporal derivatives and quadratic terms for each [@confounds_satterthwaite_2013]. Frames that exceeded a threshold of 0.9 mm FD were annotated as motion outliers. Each dataset underwent spatial smoothing with a Gaussian kernel of 6mm FWHM in SPM12.

**Appendix B -** **Supplementary analyses**

Attrition bias Follow-Up: accounting for selection and attrition biases, follow-up adherence was not explained by Group (X^2^=2.54, *p*=0.13), amyg-EFP-NF success (X^2^=2.17, *p*=0.2) or initial PCL-5 scores (*t*(51) =-1.46, *p*=0.25).

rt-fMRI-NF success analysis: Additional analysis included utilizing the recently suggested criterion for successful rt-fMRI modulation (37) defined as an average [amygdala](https://www.sciencedirect.com/topics/neuroscience/amygdala) activity of greater than zero for regulate vs baseline. This analysis showed no differences between groups in the first NF cycle pre-NF session ( *χ2* (1) = 0.12, *p* = .72), but did demonstrate a difference post-NF (*χ2* (1) = 5.788, *p* = <.05, *phi*=.42) showing higher rates of successful cycles (78%) compared to control (33%). Finally, in our secondary and unpowered analysis no significant association was found between Amg-EFP signal modulation and clinical change (reflected by delta pre-post NF total CAPS-5 scores) (r(38)=0.1 *p*=0.26).
